# Quercetin Impact in Pancreatic Cancer: An Overview on Its Therapeutic Effects

**DOI:** 10.1155/2021/4393266

**Published:** 2021-11-03

**Authors:** Parina Asgharian, Abbas Pirpour Tazehkand, Saiedeh Razi Soofiyani, Kamran Hosseini, Miquel Martorell, Vahideh Tarhriz, Hossein Ahangari, Natália Cruz-Martins, Javad Sharifi-Rad, Zainab M. Almarhoon, Alibek Ydyrys, Ablaikhanova Nurzhanyat, Arailym Yessenbekova, William C. Cho

**Affiliations:** ^1^Drug Applied Research Center, Tabriz University of Medical Sciences, Tabriz, Iran; ^2^Department of Pharmacognosy, Faculty of Pharmacy, Tabriz University of Medical Sciences, Tabriz, Iran; ^3^Department of Biochemistry and Clinical Laboratories, Faculty of Medicine, Tabriz University of Medical Sciences, Tabriz, Iran; ^4^Clinical Research Development Unit of Sina Educational, Research and Treatment Center, Tabriz University of Medical Sciences, Tabriz, Iran; ^5^Molecular Medicine Research Center, Biomedicine Institute, Tabriz University of Medical Sciences, Tabriz, Iran; ^6^Student Research Committee, Shiraz University of Medical Sciences, Shiraz, Iran; ^7^Department of Molecular Medicine, Faculty of Advanced Medical Sciences and Technologies, Shiraz University of Medical Sciences, Shiraz, Iran; ^8^Department of Nutrition and Dietetics, Faculty of Pharmacy and Centre for Healthy Living, University of Concepción, 4070386 Concepción, Chile; ^9^Department of Food Science and Technology, Faculty of Nutrition and Food Science, Tabriz University of Medical Sciences, Tabriz, Iran; ^10^Department of Biomedicine, Faculty of Medicine, University of Porto, Alameda Prof. Hernâni Monteiro, Porto, Portugal; ^11^Institute for Research and Innovation in Health (i3S), University of Porto, Porto, Portugal; ^12^Institute of Research and Advanced Training in Health Sciences and Technologies (CESPU), Rua Central de Gandra, 1317, 4585-116 Gandra PRD, Portugal; ^13^Facultad de Medicina, Universidad del Azuay, Cuenca, Ecuador; ^14^Department of Chemistry, College of Science, King Saud University, P. O. Box 2455, Riyadh 11451, Saudi Arabia; ^15^Biomedical Research Centre, Al-Farabi Kazakh National University, Al-Farabi Av. 71, 050040 Almaty, Kazakhstan; ^16^Department of Biophysics, Biomedicine and Neuroscience, Al-Farabi Kazakh National University, Al-Farabi Av. 71, 050040 Almaty, Kazakhstan; ^17^Department of Clinical Oncology, Queen Elizabeth Hospital, Kowloon, Hong Kong, China

## Abstract

Pancreatic cancer (PC) is a lethal malignancy cancer, and its mortality rates have been increasing worldwide. Diagnosis of this cancer is complicated, as it does not often present symptoms, and most patients present an irremediable tumor having a 5-year survival rate after diagnosis. Regarding treatment, many concerns have also been raised, as most tumors are found at advanced stages. At present, anticancer compounds-rich foods have been utilized to control PC. Among such bioactive molecules, flavonoid compounds have shown excellent anticancer abilities, such as quercetin, which has been used as an adjunctive or alternative drug to PC treatment by inhibitory or stimulatory biological mechanisms including autophagy, apoptosis, cell growth reduction or inhibition, EMT, oxidative stress, and enhancing sensitivity to chemotherapy agents. The recognition that this natural product has beneficial effects on cancer treatment has boosted the researchers' interest towards more extensive studies to use herbal medicine for anticancer purposes. In addition, due to the expensive cost and high rate of side effects of anticancer drugs, attempts have been made to use quercetin but also other flavonoids for preventing and treating PC. Based on related studies, it has been found that the quercetin compound has significant effect on cancerous cell lines as well as animal models. Therefore, it can be used as a supplementary drug to treat a variety of cancers, particularly pancreatic cancer. This review is aimed at discussing the therapeutic effects of quercetin by targeting the molecular signaling pathway and identifying antigrowth, cell proliferation, antioxidative stress, EMT, induction of apoptotic, and autophagic features.

## 1. Introduction

Pancreatic cancer (PC) is an increasingly common cancer of the gastrointestinal tract (GIT), with survival rates less than 5% at 5 years after diagnosis, and about 50% of all patients die over 6 months of diagnosis. According to estimations in the United States, PC will become the second most common cause of cancer death in the next twenty to thirty years. However, patients' prognosis with localized and respectable tumors remains poor with only 20% survival rate after surgery [1]. In addition, in line with GLOBOCAN 2018 assessments, PC which accounts for approximately 459,000 new cases and 432,000 deaths is the seventh leading cause of global cancer death [2]. In Europe, it is assumed that PC will quickly exceed breast cancer as the third cause of cancer death after colorectal and lung cancers [3].

PC is characterized as a tumor of the exocrine pancreas and ductal adenocarcinoma; however, a minor subset of patients also represents neuroendocrine tumors. Indeed, pancreatic intraepithelial neoplasia or precursor lesions are the operative factors in the acquisition of genetic shifts which trigger discernible pancreatic ductal adenocarcinoma (PDA) [4]. Unfortunately, PC symptoms do not begin up to the advanced stages of cancer and are usually vague, including nausea, vomiting, severe abdominal pain, and weight loss. Besides, previous studies revealed that type 2 diabetes, family history, obesity, and tobacco usage are the major risk factors for PC [1–5]. Therefore, studies are headed for PC prevention. A broad range of recent studies have explored the anticancer features of phytochemicals and have indicated that polyphenols, flavonoids, and flavones can be occupied against diverse types of cancers [6]. Flavonoids are secondary metabolites of plants with pharmacological activities (Table 1). Hence, fruits or vegetables, such as cocoa and coffee, are valuable sources of flavonoids [7, 8]. Based on chemical structure, oxidation degree, and unsaturation of linking chain, flavonoids are categorized into 6 main classes: isoflavonoids, flavones, flavanols, flavanones, flavonols, and anthocyanidins [9]. Quercetin and kaempferol are some of the most frequently found flavonols [10]. Quercetin (C_15_H_10_O_7_) is called by IUPAC (International Union of Pure and Applied Chemistry) as follows: 3,3,4,5,7-pentahydroxyflavone and 2-(3,4-dihydroxyphenyl)-3,5,7-trihydroxychromen-4-one [11]. It has been documented that quercetin offers antifungal, antioxidant, cytotoxic, hepatoprotective, and anticancer activities [12]. Specifically, both quercetin and its derivatives can prevent cancer-related diseases by regulating cellular signaling pathways. However, the anti-inflammatory and antioxidant properties of quercetin are the main factors for its activity as cell cycle inhibitors, and the apoptosis-inducing effect of quercetin has a key anticancer role [13, 14]. Noteworthy, quercetin is a general phytochemical in the regular dietary program of people worldwide since it can be widely found in daily foods, like tea, coffee, different vegetables, nuts, and fruits [15]. Quercetin and its derivatives pose biological inhibitory effects on the progression of the cancerous cell cycle; therefore, the metabolic pathways of quercetin are deemed as a significant factor in the plants' adaptive reaction. A number of recent studies have focused on the quercetin content of fruits and vegetables for its therapeutic purposes [16–19]. Additionally, as mentioned by Harwood et al. [20], commercially accessible quercetin can be consumed orally at a dose of 1 g daily, which is absorbed up to 60% and safe enough. In this sense, this review aims to discuss the anticancer properties of quercetin against PC, considering its low cost in comparison to synthetic drugs. In addition, the latest trends on quercetin features and their molecular mechanisms in cancer therapy are also summarized. Therefore, different research studies have analyzed the probable mechanisms through which quercetin exerts its antitumor effects against pancreatic cancer cells. Since there is not any review article on this subject based on our searches, we aimed to discuss the therapeutic effects of quercetin against pancreatic cancer cells for the first time.

## 2. Pancreatic Cancer (PC)

Currently with an average 5-year survival rate, PC is estimated to be the second cause of cancer-related deaths by 2030 in the United States [21–23]. The possibility of developing PC is about 1.5% in both genders [23], despite it mainly occurs in elderly, between 70 and 80 years, mostly in unlocalized and incurable forms [21, 24]. PC often remains undetectable until it transforms into a metastatic tumor [25]. While the etiology of PC has not been completely understood, several genetic and environmental risk factors are known to increase the risk, including smoking, obesity, diets rich in animal fat, cystic fibrosis, and genetic predispositions [26]. According to Huang et al. [27], the highest incidence and mortality of PC are in countries with very high human development index or age-standardized rates (ASRs) or the countries with a higher prevalence of alcohol drinking, smoking, hypertension, physical inactivity, obesity, and high cholesterol. The highest incidence rates of PC were reported in Western Europe (ASR, 8.3), North America (ASR, 7.6), and Central and Eastern Europe (ASR, 7.5). The incidence of PC has men to women ratio of 1.4 : 1.0. More detailed information about the PC incidence and mortality based on the region and sex are presented in the main reference [27].

An earlier diagnosis would be very helpful in the successful treatment of this malignancy, despite the scarce presence of symptoms among individuals. Regarding treatment, surgery, chemotherapy, and radiotherapy are the most common therapeutic strategies applied for PC treatment. Actually, the standard course of treatment is surgery following adjuvant therapy; however, the recurrence of 70-80% of resected tumors ultimately occurs. Patients who are eligible for surgical resection comprise merely almost 10-15% of all patients with advanced PC. With the majority of patients being diagnosed at later stages, chemotherapy remains as only treatment option for PC. 5-Flourouracil (5-FU) and gemcitabine (GMC), alone or in combination with radiation, are the standard chemotherapy regimen for PC's treatment, even though the response rate is usually below 31%. GMC has some advantages over 5-FU, such as the ability to relieve most disease's symptoms and having a modest survival advantage; however, it could not extend the average survival rate much beyond 6 months, like other chemotherapeutics [28, 29]. Hence, with the limited success of current standard therapies, the search for new and effective treatment strategies and agents is urgently needed.

## 3. Naturally Occurring Phytochemicals for Anticancer Purposes

Various observational and prospective studies have revealed an indirect association between fruit and vegetable consumption with the occurrence of some cancers and the great potential of natural compounds to change the natural history of carcinogenesis [30–32]. Plants with some bioactive nonnutrient compounds isolated, characterized, and identified as phytochemicals have been ever more searched for their ability to treat different diseases, especially cancer [33–41]. It seems that natural products still hold out the best options to find effective novel components in the treatment of human diseases [42]. In addition, the development of scientific technologies such as genome mining, genetic engineering, and using of nanoparticles as carriers [43] improve the discovery of new drugs in cancer therapy [44]. The word ‘phytochemical' refers to plant (phyto in Greek) chemicals. Many of these phytochemicals could regulate a wide range of cellular signaling pathways which are involved in oxidative stress, growth, proliferation, differentiation, and death [37, 45–48]. For example, they exhibit antioxidant properties by affecting Nrf2-Keap1 pathway, where upon activation, Nrf2 translocate into the nucleus, binds to ARE (antioxidant response elements) or EpREs (electrophile response elements) and increases the expression of ATP-dependent drug efflux pumps, detoxification enzymes, and endogenous antioxidants [49]. These events eventually lead to the protection of cells against ROS (reactive oxygen species) [50, 51]. Phytochemicals could also suppress tumor progression and induce apoptosis in pre-neoplastic or neoplastic cells by affecting cell cycle, JAK-STAT, NF-*κ*B, and cytochrome C signaling pathways [52, 53]. One of the phytochemicals is garcinol, a Polyisoprenylated Benzophenone that can inhibit STAT-3 pathway by suppressing the upstream kinases (c-Src, JAK1, and JAK2) in HNSCC cells. Garcinol also inhibits NF-*κ*B activation by the suppression of TGF-*β* and inhibitor of I*κ*B kinase (IKK) activation in HNSCC cells [54]. In addition, Li et al. showed that garcinol prevents the growth of human HNSCC xenograft tumors in male athymic nu/nu mice [54]. It can be concluded that garcinol has potential antitumor effects in head and neck carcinoma through the suppression of multiple proinflammatory cascades. Activator protein 1 (AP-1) as a key transcription factor in the control of several cellular processes is involved in inflammatory disorders and cancer. Several natural compounds such as kaempferide, resveratrol, apigenin, isorhamnetin, citrifolinoside A, viscolin, curcumin, and quercetin can modulate AP-1-associated signaling pathways for cancer prevention and intervention [55].

**Figure 1:**
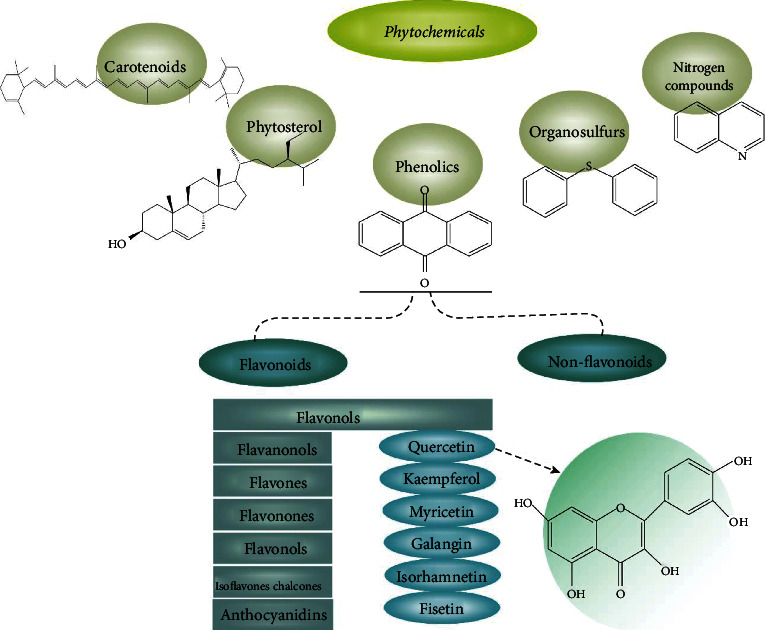
Classified phytochemicals with anticancer potential and their chemical structure.

Among the plethora of biologically active phytochemicals with anticancer potential, they are chemically categorized into phenolics, carotenoids, phytosterols, organosulfur compounds, and nitrogen-containing compounds [56, 57]. Phenolics are structurally characterized with one (phenolic acids) or more (polyphenols) aromatic rings with one or more hydroxyl (OH) groups [58]. Phenolic compounds can be divided into flavonoids and nonflavonoids [59]. Flavonoids, including glycosides, aglycone, and methylated derivatives, comprise half of phenolic compounds [60]; flavonoids are subgrouped into flavones, flavanones, flavanonols, flavanols, flavonols, isoflavones, chalcones, and anthocyanidins [61, 62]. Nonflavonoids also have several subgroups which include stilbenes, phenolic acids, lignans, coumarins, and tannins [63] (Figure 1).

## 4. Flavonoids and Anticancer Effects: Key Focus on Quercetin

Belonging to the class of polyphenolic flavonoid compounds and the subclass of flavonols, quercetin is ubiquitous in daily foods, including various plants, vegetables, nuts, seeds, fruits, tea, and red wine [64, 65]. However, fruits and plants are being studied as promising sources of quercetin [17, 66–68]. Quercetin comprises the characteristic structure of flavonoids (backbone C6-C3-C6) in which two benzene rings are bonded by a 3-carbone heterocyclic pyrone [69, 70]. Quercetin has two antioxidant pharmacophores in this structure, which allow to act as a free radical scavenging agent and join to transitional metal ions [69]. The ideal arrangement of the catechol and the OH group at C_3_, the position in quercetin structure, also adds to its free radical scavenging ability [69, 71]. The replacement of its various OH groups grants quercetin different biochemical and pharmacological functions [72]. It has been estimated that the average daily intake of quercetin could be about 25 mg [20]. The bioavailability of quercetin relies on its metabolic form in the food [73]. Quercetin may be found as free or aglycone state and conjugated forms, in which it interacts with several molecules, including lipids, carbohydrates, alcohols, and sulfate groups to form its derivatives, including prenylated quercetin, quercetin ethers, quercetin glycoside, and quercetin sulfate [72]. In plants, the form of quercetin is quercetin glucosides (quercetin-glucose conjugates). Quercetin glucosides undergo hydrolysis to form quercetin aglycone following the absorption in the apical membrane of the enterocytes. Then, enterocytic transferases metabolize quercetin aglycone to the glucuronidated, sulfonylated, and methylated forms [73]. These quercetin metabolites when transported to the liver undergo other conjugation processes to generate Que-3-glucuronide and quercetin-3′-sulfate [73–75]. The peak plasma concentration of quercetin varies from 3.5 to 5.0 *μ*M once being absorbed in the form of glucosides. However, its peak plasma concentration is less than 0.33 *μ*M when absorbed in the unconjugated form, showing less efficient absorption [76].

Quercetin has numerous benefits on human health, including anticancer, antioxidant, antidiabetic, antiulcer, anti-inflammatory, antiviral, antiallergic, antihypertension and anti-infection, cardioprotective, gastroprotective, and immune-modulatory effects [69, 77]. With the specific impact on tumor cells and without any impact on normal and nontransformed ones, quercetin has fascinated many researchers to investigate its potential as an adjuvant to suppress oxidative stress, proliferation, and metastasis [78]. Several studies showed the inhibitory impacts of quercetin against pancreatic, colorectal, prostate, lung, ovarian, nasopharyngeal, breast, and kidney cancers [79–85]. A number of recent clinical studies have scrutinized quercetin's effect on PC. In this regard, Liu et al. [86] have explored the anticancer effects and mechanistic actions of quercetin in GMC-resistant cancer cells. In this survey, BxPC-3, PANC-1 and HepG2, and Huh-7 cell lines were studied. Proliferation assays presented that quercetin had cytotoxic effects on GMC-resistant cell lines including HepG2 and PANC-1, and flow cytometric analysis specified a noteworthy proapoptotic effect on these cell lines. GMC treatment, along with quercetin, caused increased anticancer effects compared with GMC alone, and quercetin led to S phase arrest in resistant cell lines. Hoca et al. [5] investigated the effect of quercetin and resveratrol on epithelial-mesenchymal transition (EMT) of CD133^+^ and CD133^−^ pancreatic cancer cells. CD133^+^ cells were obtained from the PANC-1 cells by the MiniMACS system. CD133^+^ and CD133^−^ PANC-1 cells were treated with different concentrations of resveratrol and quercetin. Immunocytochemistry tests using antibodies such as TNF-*α*, ACTA-2, N-cadherin, IL-1*β*, and vimentin were applied for assessing the anticancer and antimetastatic properties of resveratrol and quercetin. Results revealed that the immunostaining intensity of CD133^+^ cells was stronger than that of CD133^−^ cells. ACTA-2, N-cadherin, and IL-1*β* immunoreactivities were significantly decreased, whereas vimentin and TNF-*α* immunoreactivities increased in quercetin treated CD133^+^ cells. In addition, quercetin was more effective than resveratrol in inhibiting metastasis. Guo et al. [87] have studied the therapeutic potential of quercetin in targeting sonic hedgehog (SHH) signaling of PDA. The effects of quercetin on the apoptosis, migration, and invasion of pancreatic cancer cells (PCCs) were evaluated in PDA xenograft mouse models. According to the results, quercetin inhibited the PCC proliferation by downregulating c-Myc expression and suppressed the EMT by reducing TGF-*β*1 level, which inhibited the PCC migration and invasion. Quercetin treatment reduced the PDA growth and metastasis in nude mouse models by decreasing SHH activity. Additionally, SHH activated TGF-*β*1/Smad2/3 signaling and stimulated EMT by inducing the expression of Snail1and Zeb2 that instigated a partial reversal of quercetin-mediated inhibition of PCC migration and invasion.

### 4.1. Molecular Mechanisms Underlying Quercetin-Mediated Effects in Cancer

#### 4.1.1. Effect in Autophagy and Apoptosis Induction

**Figure 2:**
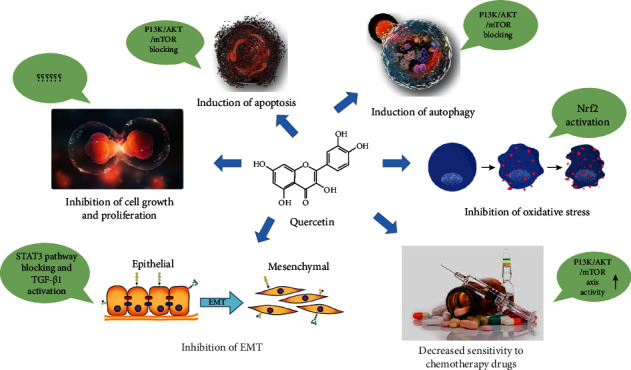
Schematic representation of the molecular structure of quercetin composition and its role in physiological conditions through signaling pathways. The flavonoid quercetin through inhibitory and stimulatory mechanisms performs functions such as inducing autophagy and apoptosis and reducing or inhibiting cell growth and proliferation, EMT, oxidative stress, and sensitivity to chemotherapy drugs.

According to Pang et al. [88], quercetin can affect CD36 and decrease the death rate of PC by facilitating the uptake of fatty acids, improving the cell adhesion, stimulating immune response, and regulating thrombospondin-1. Furthermore, previous trends indicated that quercetin has proapoptotic activity in suppressing Bcl-2 protein and in upregulating the p53 gene; however, inhibition of Bcl-2 transcription could prevent the tumors development [12]. In an illustrative study, Serri et al. [89], have investigated the effect of GMC with biodegradable nanoparticles (NPs) loaded within quercetin on PC cell lines. The manufactured NPs decorated with hyaluronic acid (HA) and loaded with quercetin and GMC presented a developed cytotoxicity on PANC-1 and Mia-PaCa-2 cell lines when compared with the bare drugs and the NPs nondecorated with HA on the surface. The results indicated that, the NPs exposing HA may enhance the anti-inflammatory activity of Que, which led to a reduction of interleukin (IL) expression levels in cell lines and preliminarily increased with lipopolysaccharides (LPS). In another survey, Lan et al. [90] showed that quercetin accelerates cell death and chemosensitivity of human PC cells. The results showed that silencing of a receptor for advanced glycation end products (RAGE) by RAGE-specific siRNA intensified the autophagy and apoptosis through suppressing PI3K/AKT/mTOR axis in MIA Paca-2 and GMC-resistant cells (MIA Paca-2 ^GMCR^ cells). Moreover, quercetin reduced RAGE expression and facilitated the apoptosis, autophagy, and chemosensitivity to GMC in MIA Paca-2 ^GMCR^ cells, which suggests that further cytotoxicity has been achieved by the addition of quercetin in treatment with GMC. Yu et al. [91] showed that quercetin initiated inhibitory activities against PATU-8988 and PANC-1 cells and reduced the release of matrix metalloproteinase (MMP). In this study, they used STAT-3 and IL-6 activation to scrutinize the effects of quercetin treatment on cell malignancy. The MMP secretion and epithelial mesenchymal transition (EMT) stimulated the STAT-3 signaling pathway, while quercetin reversed IL-6-induced EMT and invasion. As main findings, this study showed that quercetin is an effective agent in PC treatment as it blocks the STAT-3 signaling pathway, leading to the suppression of EMT and metastasis. In addition, Nwaeburu et al. [92] have explored the effect of quercetin on miRNA expression in PC cells and concluded that quercetin treatment induced the expression of miR-200b-3p in AsPC1 cell lines, which has a crucial role in the irregular division of PDA cells by notch signaling regulation (Figure 2).

#### 4.1.2. Effect in Proliferation and Cell Growth

Inhibition of PC cell proliferation could signify a distinct mechanism of anticancer effects of quercetin (Table 2). In this way, Pham et al. [93] studied the effect of quercetin on dysregulated epigenetic readers, including bromodomain and extraterminal domain (BET) proteins, in *in vitro* and xenograft models of PC. According to the results, after treatment with BET inhibitors and quercetin the proliferation and sphere-forming ability of cancer cells was reduced, and apoptosis stimulated. In addition, quercetin diminished the nuclear protein hnRNPA1 which control mRNA translation and export of antiapoptotic proteins, *in vivo* and increased the BET inhibitors effects at suppressing cells proliferation and tumor growth. In another study, Nwaeburu et al. [94] explored the quercetin influence on PC cells proliferation by the activation of Notch-inhibitor Numbl as let-7c target gene. *In vivo* xenotransplantation of PDA cell and following IV injection of let-7c provoked a noteworthy reduction of tumor mass in the fertilized chick egg model. Immunohistochemistry analysis demonstrated that let-7c upregulated the Numbl and reserved Notch and progression markers. The findings illustrate that Que-induced let-7c declines cancer cell divisions and tumor growth.

#### 4.1.3. Effect in Oxidative Stress

Redox homeostasis is very important for cell function and ROS have an essential role in cell signaling. However, the disturbance in the antioxidant system could lead to excessive intracellular ROS levels, such as hydroxy free radicals and H_2_O_2_ [95]. The excessive intracellular levels of ROS result in oxidative damages to many biological macromolecules which includes lipids, proteins, and genetic material, giving rise to pathological conditions, like cancer, inflammation, atherosclerosis, angiogenesis, as well as aging [96–100]. Therefore, helping cells to keep redox homeostasis is of great value, which can be provided by consuming natural nutritional components, such as Que.

With the numerous OH groups and conjugated *π* orbitals allowing it to donate hydrogen or electrons, and thus scavenge superoxide anion (•O_2_−) and H_2_O_2_. Quercetin is regarded as outstanding free-radical scavenging antioxidant [101]. Quercetin could generate the semi-quinone radical and H_2_O_2_ by reaction with •O_2−_, while also decreases H_2_O_2_ levels in the presence of peroxidases and keeps cells safe against H_2_O_2_ damages [64]. The semi-quinone is one of potentially harmful reactive oxidation products and undertakes a second oxidation reaction with Que, producing additional quinone (Que-Quinone; QQ) [64]. QQ is held culprit for lipid peroxidation as well as protein and DNA damages with higher affinity to react with lipids, proteins, and DNA [64, 102]. QQ with high reactivity towards thiols could arylate protein thiols, impairing several vital enzymes; however, it generates relatively stable glutathione (GSH)-oxidized adducts including 8-glutathionyl-quercetin (8-GSQ), 6-glutathionyl-quercetin (6-GSQ), and 2′-glutathionyl-quercetin (2′-GSQ) when reduced GSH exists [103, 104]; this reaction is reversible and glutathionyl-quercetin adducts could be constantly disassociated into QQ and GSH [105]. Consequently, high GSH concentrations within cells, oxidized quercetin forms GSQ by reaction with GSH, neutralizing the toxicity of QQ. Yet, oxidized quercetin reacts with protein thiols while lower concentrations of GSH exist within cells, showing the prooxidant effect of quercetin [105, 106]. Therefore, the GSH concentration within cells determines whether the antioxidant effect of quercetin could prevail over its prooxidant effect. Indeed, high levels of GSH limit quercetin cytotoxicity and permit it to show its antioxidant activity but not prooxidant activity [107]. Besides, it has been shown that quercetin induces GSH synthesis [108, 109]. Moreover, quercetin also exerts antioxidant activity by activating the nuclear factor erythroid 2-related factor 2 (Nrf2) as well as its downstream targets, which are vital for maintaining cell redox hemostasis [110, 111].

#### 4.1.4. Effect in Epithelial-to-Mesenchymal Transition (EMT)

A physiological process, epithelial-to-mesenchymal transition (EMT), has an key function in mammalian embryonic development and cell and tissue balance; however, it has also an important role in tumorigenesis and tumor progression [112]. During EMT, epithelial cells undergo some changes including losing cellular polarity, disabling junctions between cells and adhesive connections, and obtaining penetration and migration capabilities [113, 114]. EMT can be monitored by protein markers, including E- and N-cadherin, Snail and Vimentin [114, 115]. Furthermore, MMPs closely related to EMT are recently introduced as EMT markers and as a predisposing factor for it, providing an appropriate condition for tumor infiltration and metastasis by degrading the extracellular matrix (ECM) and basement membrane (BM) nearby the tumor exterior [116, 117]. The impact of quercetin on EMT in PC cells have been investigated by a few studies. In one study, it has been shown that quercetin treatment could decrease EMT and MMP secretion in PATU-8688 PC cell line [91]. Quercetin reduced mRNA and protein expressions level of N-cadherin, Slug, Vimentin Zeb1, Twist, and Snail, indicating the potential of quercetin to reverse the EMT process; however, it increased E-cadherin expression [91]. Quercetin also inhibited MMP2 and MMP7 protein expressions [91]. Besides, it has been indicated that quercetin exerted its inhibitory impacts on EMT, invasion, and metastasis in PC cells through suppressing the STAT-3 signaling pathway [91]. Another study showed that quercetin repressed EMT by suppressing SHH and TGF-*β*/Smad signaling pathways, involved in promoting EMT by the induction of Zeb2 and Snail1 expressions [87]. Quercetin downregulated Vim (encoding vimentin) and Acta2 (encoding *α*-SMA) gene expressions, and upregulated Cdh1 (encoding E-cadherin) gene expression in PANC-1 and Patu8988 cells; upon quercetin treatment, the protein levels of type I collagen, N-cadherin, *α*-SMA and vimentin were reduced,; however, the protein level of E-cadherin was increased in cells [87]. Quercetin decreased TGF-*β*1 expression and that of EMT-TFs (EMT-inducing transcription factors) Snail1 and Zeb2 [87]. EMT-TFs (Snail1 and Zeb2) are the key downstream target of TGF-*β*1/Smad2/3 signaling pathway suppressing E-cadherin expression [118, 119]. Furthermore, the nuclear translocation and phosphorylation of Smad2 and Smad3 were also suppressed by quercetin [87]. It has been reported that upon activation by TGF-*β*1 and forming heteromeric complexes with Smad4, Smad2 and Smad3 translocate to the nucleus and induce EMT-TFs' expression [120]. It has also been indicated that quercetin may inhibit EMT in PC stem cells by suppressing the expression of N-cadherin [5]. Quercetin downregulated Twist2 expression, a protein involved in EMT, in PC stem cells [121], suggesting EMT inhibition by quercetin [122].

#### 4.1.5. Effect in Chemo-Sensitivity

By the improved effectiveness in combination with other dietary agents, quercetin has been investigated as a promising adjuvant to increase the effectiveness of numerous chemotherapeutics [122, 123]. Lan, Chen, Kuo, Lu and Yen [90] showed that quercetin may decline cell viability, promote autophagy, and increase apoptosis by suppressing receptors for advanced glycation end products (RAGE) in GMC-resistant PC cells, with a greater impact once accompanied with GMC. The results revealed that RAGE silencing promoted GMC-induced cytotoxicity in MIA Paca-2 and MIA Paca-2 ^GEMR^ cell via the PI3K/AKT/mTOR axis [90]. As RAGE silencing, quercetin reduced the expression of RAGE, which led to cell cycle arrest, apoptosis, autophagy, and promoted GEM efficacy in MIA Paca-2 GEMR cells [90], proposing quercetin quercetin as enhancer of chemotherapy efficacy of drugs against PC. In another study, quercetin promoted tumor necrosis factor-related apoptosis-inducing ligand (TRAIL)-induced apoptosis in TRAIL-resistant PC cells [124], and decreased cellular FLICE-like inhibitory protein (cFLIP) expression, while activated c-Jun N-terminal kinase (JNK), leading to the proteasomal degradation of cFLIP and eventually making PC cells more susceptible to TRAIL-induced apoptosis [124]. It has also been reported that quercetin decreased the viability of PC cell lines including PANC-1, MiaPaCa-2, and BxPC-3 [125, 126]. Once combined with other chemotherapeutics, such as GMC or 5-FU, quercetin could affect chemotherapy efficacy depending on cell lines applied, either to suppress proliferation of or have no impact on cancer cells [125, 126]. Borska et al. [127] indicated that quercetin induced apoptosis and suppressed cell proliferation in both daunorubicin sensitive EPP85-181P and resistant EPP85-181RDB PC cell lines. Quercetin had synergistic effects with daunorubicin in both sensitive and resistant cells [127]. They also showed that quercetin treatment could decrease P-glycoprotein expression [128].

## 5. Conclusion

Food consumption combined with therapeutic agents has been considered a key for the successful treatment of several diseases, including cancer. Conventional therapies like natural components besides other therapeutic methods due to their lower cost and side effects have been increasingly considered by researchers. Specifically, quercetin exerts an anticancer effect against PC cancer cells by mediating apoptosis, but recent studies have also indicated that quercetin affects various signal transduction pathways to reduce cancer progression. Quercetin suppresses the expression of N-cadherin, MMP-9, STAT-3 signaling pathways and potentially inhibits EMT, invasion, and metastasis. Quercetin enhances gemcitabine chemosensitivity in pancreatic cancer cells through the inhibitory effect on RAGE expression. Meanwhile, it has wide accessibility, efficacy and low toxicity comparing with other studied compounds, make it an appealing agent in cancer treatment. More recently, quercetin has been introduced and applied as a promising drug in the treatment of various cancers alone or in combination with other chemotherapeutic agents. Future well-designed clinical studies are needed to help the scientists to evaluate the safety and potential of quercetin against PC.

## Figures and Tables

**Table 1:** A number of pharmacological activities of quercetin reported in recent studies.

Dose	Model	Administration	Activity	Ref.
25 *μ*M	MCF-7 and MDA-MB-231	Direct treatment	Apoptosis induction and anticancer effect	[129]
20 *μ*M	Human umbilical vein endothelial cells (HUVECs)	Direct treatment	Autophagy and promoted cell survival	[130]
5.7 *μ*M (DPPH assay)	Erythrocytes	Direct treatment	ROS and free radical scavenging activity	[131]
100 mg kg^−1^	C57BL/6J mice on ethanol-containing Lieber De Carli liquid diets	Gavage	Suppressed autophagic flux, decreased liver injury by ethanol consumption	[132]
100 mg kg^−1^	*Streptococcus suis* infected mice	Subcutaneous	Antimicrobial effect against *Streptococcus suis*	[133]
100 mg kg^−1^	Chronic ethanol feeding C57BL/6J mice	Oral	Decreased fat accumulation in liver (ethanol induced)	[134]
30 mg kg^−1^	STZ-induced diabetic rats	Intraperitoneal	Higher insulin levels, improved dyslipidemia, reduced serum blood glucose levels, decreased oxidative stress	[135]
100-200 mg kg^−1^	STZ-induced diabetic Wistar rats	Oral	Controlled insulin resistance, reduced blood sugar, pancreatic cells protection	[136]
5-20 mg kg^−1^	STZ-induced diabetic rats	Oral	Controlled body weight and blood glucose, performance in the Morris water test	[137]
40 mg kg^−1^	STZ-induced diabetic mice in the Morris water maze task	Oral	Enhanced the time spent by mice in the target quadrant in the Morris water maze task	[138]

**Table 2:** Anticancer effects of quercetin against PC.

Dose	*In vitro/in vivo*	Cell line	Effective mechanism	Ref.
100 *μ*M and 75 mg kg^−1^	*In vivo* and *in vitro*	PANC-1 and Patu8988	EMT suppression by reducing TGF-*β*1 level, inhibition of growth, invasion, and migration of cells, apoptosis of cancer cells by antagonizing TGF-*β*/Smad and SHH signaling pathways	[87]
20 *μ*M	*In vitro*	Mia-PaCa-2 and PANC-1	Reduced IL-6 and IL-8 expressions and enhanced cytotoxicity against Mia-PaCa-2 and PANC-1 cell lines	[89]
100 *μ*M	*In vitro*	PANC-1	Reduced immunoreactivities such as ACTA-2, IL-1*β*, and N-cadherin, increased TNF-*α* and vimentin, prevention of EMT	[139]
20 *μ*M and 40 mg kg^−1^	*In vivo* and *in vitro*	PDAC	Improved effects of BET inhibitors at suppressing tumor development and reduced hnRNPA1 *in vivo*	[93]
50-200 *μ*M	*In vitro*	MIA Paca-2, BxPC-3, AsPC-1, HPAC and PANC-1	Quercetin showed a RAGE silencing like effect that attenuate RAGE expression to accelerate apoptosis, autophagy, and chemosensitivity of MIA Paca-2 ^GEMR^ cells	[90]
20-80 *μ*M	*In vitro*	PANC-1 and PATU-8988	Quercetin reversed IL-6-induced EMT by the stimulation of the STAT3 signaling pathway and prevented the migration	[91]
50 *μ*M	*In vivo*	AsPC-1 and PANC-1	Upregulation of miR-200b-3p that promoted the Notch signaling pathway of daughter cells to turn into symmetric	[92]
50 *μ*M	*In vitro*	AsPC-1, CRL-4023, and PANC-1	Notch inhibition by quercetin-induced let-7c and marker progression, upregulation of Numbl, and tumor development reduction	[94]
100 nM	*In vitro*	CFPAC-1 and SNU-213	Suppressed TGF-*β*- and VEGF-A-induced migratory activity induced at low dosages in CFPAC-1, but not in bFGF-activated SNU-213 cells	[140]

## Data Availability

The data used to support the findings of this study are available from the corresponding author upon request.
